# Taxonomy, palynology and distribution notes of seven species of *Passiflora* L. (Passifloraceae s.s.) newly recorded from Brazil

**DOI:** 10.3897/phytokeys.95.22342

**Published:** 2018-01-30

**Authors:** Ana Carolina Mezzonato-Pires, Michaele Alvim Milward-de-Azevedo, Cláudia Barbieri Ferreira Mendonça, Vania Gonçalves-Esteves

**Affiliations:** 1 Universidade Federal do Rio de Janeiro, Museu Nacional, Departamento de Botânica, Quinta da Boa Vista, São Cristovão, CEP: 20940-040, Rio de Janeiro, RJ, Brasil; 2 Universidade Federal Rural do Rio de Janeiro, Instituto Três Rios, Departamento de Ciências do Meio Ambiente, Avenida Prefeito Alberto da Silva Lavinas 1847, Centro, CEP: 25802-100, Três Rios, RJ, Brasil

**Keywords:** Amazon basin, *Astrophea*, new records, Passifloraceae, pollen, taxonomy

## Abstract

Passiflora subgenus Astrophea is one of the five recognised subgenera of *Passiflora*. Brazil presents ca. 26 species of this subgenus with the majority distributed in the Amazon Basin. During the ongoing taxonomic revision of the Brazilian species of subg. Astrophea, seven species previously unknown for the country were recorded: *Passiflora amoena*, *P. fuchsiiflora*, *P. jussieui*, *P. ovata*, *P. plumosa*, *P. quelchii*, and *P. tessmannii*. The new records expand the species distribution ranges, especially for *P. plumosa*, which was exclusively known from its type locality and *P. quelchii*, which was known only for southeastern Guyana. The authors provide taxonomic and palynological descriptions, distribution maps and illustrations for these species, in the hope that the knowledge and understanding of Brazilian Passifloraceae s.s. will be improved.

## Introduction

*Passiflora* L. is by far the largest genus in the Passifloraceae *s.s.*, with ca. 400 accepted species and a pantropical distribution (Feuillet and MacDougal 2007). The genus is currently divided into five subgenera: Passiflora subg. Astrophea (DC) Mast., P. subg. Deidamioides (Harms) Killip, P. subg. Decaloba (DC.) Rchb., P. subg. Passiflora, and P. subg. Tetrapathea (DC.) P.S.Green (Feuillet and MacDougal 2003, Krosnick et al. 2009).

Passiflora subg. Astrophea is a mainly neotropical group, with ca. 60 accepted species. The diversity of the subgenus is concentrated in lowland forest formations in the Amazon and the Guyana Shield regions (Ulmer and MacDougal 2004). Feuillet and MacDougal (2003) divided P. subg. Astrophea into two supersections: the supersection Astrophea, with three sections (*Astrophea*, *Capreolata* J.M. MacDougal & Feuillet and *Leptopoda* Killip ex Feuillet & Cremers) and the supersection Pseudoastrophea (Harms) Feuillet & MacDougal, with two sections (*Pseudoastrophea* (Harms) Killip and *Botryastrophea* (Harms) Killip) and two series (*Botryastrophea* (Harms) J.M. MacDougal & Feuillet and *Carneae* Feuillet). The subgenus is characterised by its well-developed woody stems generally reaching the canopy way above 30 m, entire leaves, the presence of two petiolar glands and diminute stipules and bracts. Difficulties in the collection of plant material due to the lianoid habit of these species are reflected in the small number of collections available in herbaria.

Brazil is the most species-rich country with ca. 26 species of P. subg. Astrophea (Flora do Brasil under construction). The majority of the Brazilian species in this subgenus are recorded for the Amazon biogeographical domain (ca. 15 species), which represents 70% of the total for the country. Colombia is the second most species-rich country with 24 accepted species, including the recently described *P. gironensis* C.Aguirre, M.Bonilla & A.Rojas (Aguirre-Morales et al. 2016). Nonetheless, the species of *Passiflora* from the Amazon domain are currently poorly understood, with P. subg. Astrophea being especially problematic.

Pollen morphology is important in Passifloraceae species delimitation. This was also confirmed for P. subg. Astrophea by Mezzonato-Pires et al. (2017), who found that amongst all pollen characters, the most significant was the sexine ornamentation.

As a result of an ongoing taxonomic revision of the Brazilian species of subgenus Astrophea, seven species were recorded for the first time in the Brazilian territory. The new records expand the distribution range for these taxa, especially for *P. plumosa*, which was exclusively known from its type locality and *P. quelchii*, which was known only from southeastern Guyana. Morphological descriptions, distribution maps and illustrations have been provided for these species, aiming to provide a better understanding of the Brazilian Passifloraceae.

## Methods

The specimens analysed were deposited in the following herbaria: HAMAB, IAN, INPA, MBM, MG, NY, R, RB, SP, SPF, UB, UEC and UPCB, acronyms following Index Herbariorum (http://sweetgum.nybg.org/science/ih/). For each species, descriptions, distribution maps, illustrations of leaves, flowers and pollen grains were provided. The pollen material was processed with the acetolysis method established by Erdtman (1952) and using light microscopy (LM) for pollen grain observations and measurements. Size of pollen grains were classified according to Erdtman (1952) as follows: very small (<10 µm), small (10–25 µm), medium (25–50 µm), large (50–100 µm), very large (100- 200 µm) and giant (> 200 µm). For scanning electron microscope (SEM) analysis observed with a Jeol JSM–6510, anthers were macerated and non-acetolysed pollen grains were sprayed on stubs covered with carbon tape (Melhem et al. 2003). For sectional classification of the species, the authors followed Radford et al. (1974) and Feuillet and MacDougal (2003). For the description of structures, the terminology used by Punt et al. (2007) and Mezzonato-Pires et al. (2017) was also used. Field photographs were only available for *P. amoena*.

## Results

**Figure 1.**
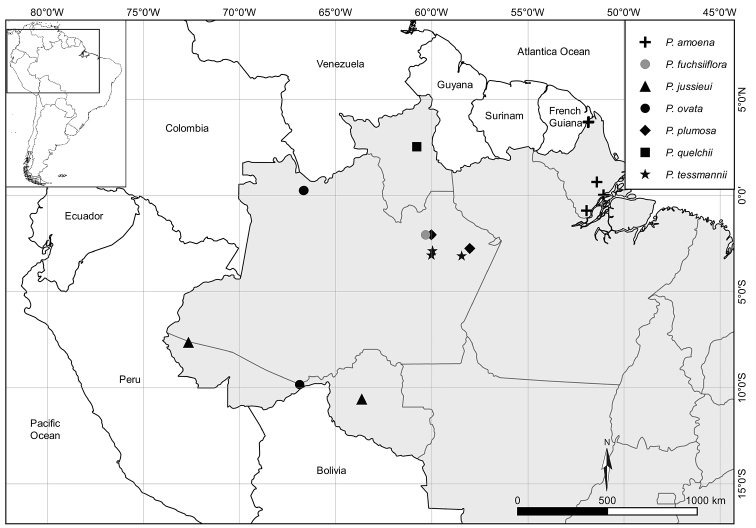
Distribution map of new plant records of species of P. subg. Astrophea for Brazil.

Seven species are recorded for the first time for Brazil: *Passiflora amoena* L.K.Escobar, *P. fuchsiiflora* Hemsl., *P. jussieui* Feuillet, *P. ovata* Jos.Martin *ex* DC., *P. plumosa* Feuillet & Cremers, *P. quelchii* N.E.Br. and *P. tessmannii* Harms. The distribution of each species in the Brazilian territory is shown in Figure 1.

### Passiflora amoena

Taxon classificationPlantaeMalpighialesPassifloraceae

L.K. Escobar, Systematic Botany 19(2): 203–205, f. 1. 1994.

**Figure 2.**
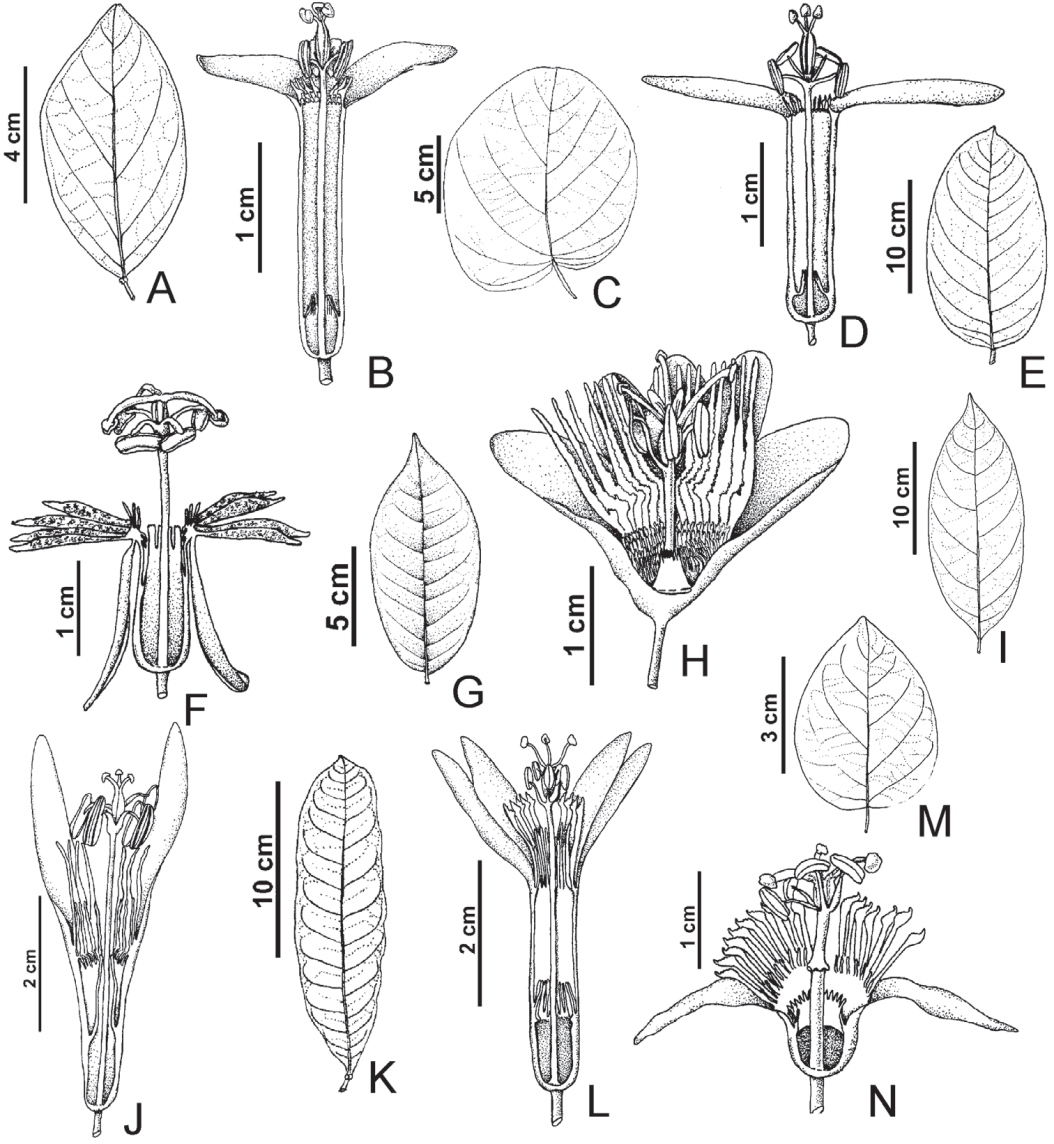
Illustrations of leaves and flowers in longitudinal section. **A, B** *Passiflora amoena* (S.V. Costa Neto et al. 2818, HAMAB) **C, D** *Passiflora fuchsiiflora* (S. Sakagawa et al. 248, INPA) **E, F** *Passiflora jussieui* (*D.C. Daly* et al. *7423*, UPCB) **G, H** *Passiflora ovata* (S. Sakagawa et al. 668, INPA) **I, J** *Passiflora plumosa* (C.A. Cid et al. 561, MG) **K, L** *Passiflora quelchii* (R.C. Forzza et al. 8321, RB) **M, N** *Passiflora tessmannii* (C.A. Sothers & E.C. Pereira 612, INPA).

**Figure 3.**
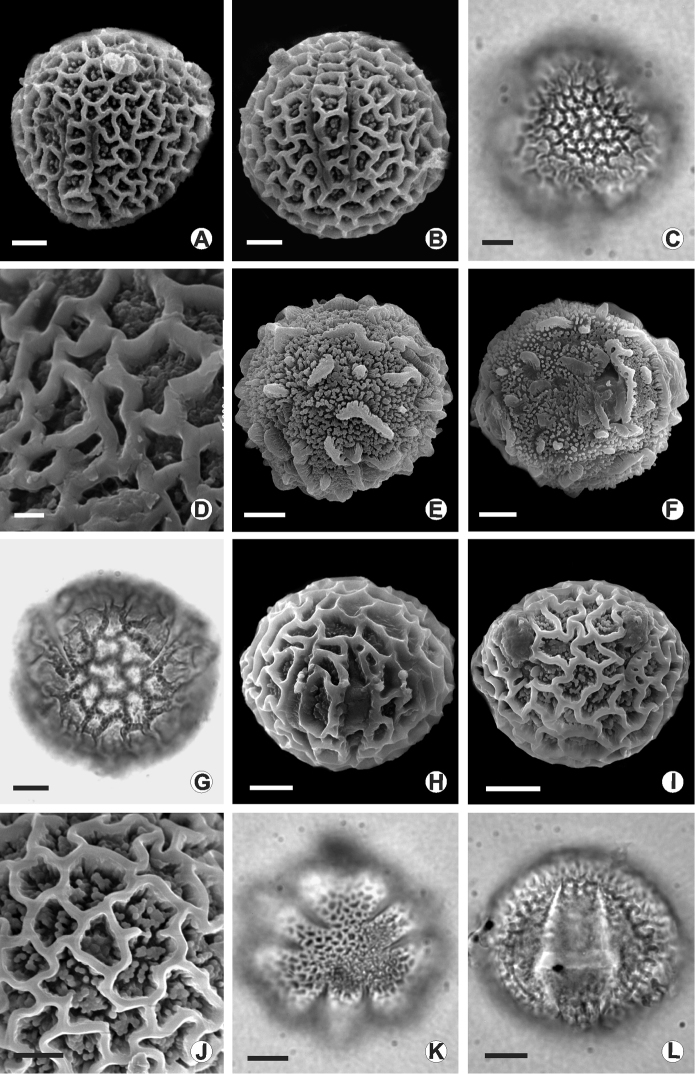
Photomicrographs and electron photomicrographs of pollen grains of the species of *Passiflora*. **A, B** *Passiflora amoena* **C, D** *Passiflora fuchsiiflora* **E, F** *Passiflora jussieui* **G, H** *Passiflora ovata* **I, J** *Passiflora quelchii* **K, L** *Passiflora tessmannii* **A, C, E, G, K** general aspect of polar view **B, F, H, I, L** general aspect of equatorial view, **D** and **J** ornamentation detail. Scale bars: 10 µm (**C, E, F, G, H, I, K, L**); 5 µm (**A, B, J**); 1 µm (**D**).

**Figure 4.**
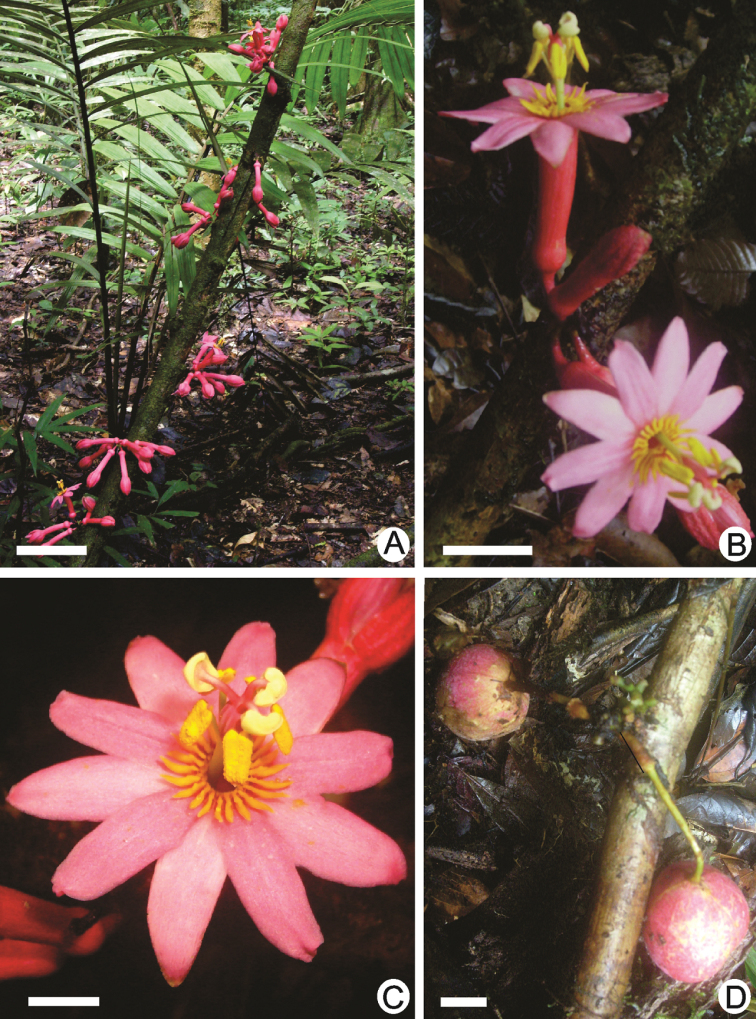
*Passiflora amoena* L.K. Escobar. **A** habit **B** inflorescence **C** flower **D** fruits. Scale bars: 10 mm (**A, C, D**); 2 mm (**B**). Photographs by S.C. Neto (S.V. Costa Neto et al. 2818, HAMAB)

Figs 2A, B , 3A, B , 4A, D

#### Descriptions.

***Lianas***; tendrils thin to robust, glabrous. ***Stipules*** not seen. ***Petioles*** with two glands on the terminal end of the adaxial side. ***Blades*** 5.6–15 × 2.7–7.9 cm, chartaceous, elliptic or ovate, apex acute-mucronate or obtuse or emarginate, base cuneate, glabrous on both sides, discolorous, abaxial side greyish-brown, adaxial side purplish-vinaceous; margins non-undulate, glandular, 2–6 glands distributed on the abaxial side of the blade; 8–14 pairs of secondary veins, arcuate. ***Bracts*** diminute, triangular. ***Flowers*** arranged in racemose inflorescences, hypanthium cylindrical; sepals oblong, dark pink; petals oblong, pink; corona with 3 series of filaments, yellow to orange-yellow, filaments of first series with dolabriform-triangular, filaments of the second series with tuberculate wavy margins; operculum straight, non-tubular, included, ligulate, apex plicate; trochlea absent on the androgynophore; ovary ellipsoid, glabrous. ***Fruits*** 6.45 × 2.24 cm, ellipsoid, glabrous.

#### Palynology.

Pollen grains medium-sized (ca. 42.8 µm), prolate spheroidal, 6-colporate, colpi long, narrow, three endoapertures lalongate (ca. 6.2 × 8.1 µm), unique for each pair of ectoaperture, sexine reticulate, heterobrochate, with muri (ca. 1.2 µm), muri simple columellate, sinuous, continuous, with perforations, without high columellae, not apparent, tectum surface mostly curved, lumina ornamented, small (ca. 6.7 µm diam.) (Fig. 3A, B).

#### Specimens examined.

**BRAZIL. Amapá**: Amapari, Cabeceiras of the Rio Amapari, on the left bank of the rio Anacuí, trail 5, 01°50'41"N, 52°44'29"W, 7 Mar 2006 [fl], M.O. Hamada et al. 151 (HAMAB, INPA, R); Clevelândia do Norte, Rio Oiapoque, along the road between Oiapoque and Clevelândia, 3°48'48"N, 51°51'38"W, 20 Jul 1960 [fr], B. Maguire, J.M. Pires and C.K. Maguire 47085 (NYBG); Igarapé Ponta-Narri, third waterfall, 08 Oct 1949, [fl], G.A. Black 49-8461 (IAN); Macapá, Colônia do Torrão, 0°2'20"N, 51°3'59"W, 29 Aug 1962 [bt, fl], J.M. Pires & P.B. Cavalcante 52667 (IAN); Oiapoque, BR156, road between Calçoene and Oiapoque, 17 km southeast of Oiapoque, ca. 3°50'35"N, 51°50'6"W, 3 Dec 1984 [fl], S.A. Mori, J. Reitsma and R. Cardoso 17157 (HAMAB); Rio Oiapoque, about 1 km west of Cachoeira Utussansain, 2°8’N, 52°55’W, 8 Sep 1960 [fl], H.S. Irwin et al. 48080 (IAN); Rio Oiapoque, upper slopes and hilltop Tipac, 3°36’N, 51°19’W, 200-250m, 15 Oct 1960 [bt, fl], H.S. Irwin 48731 (IAN); Porto Grande, Floresta Nacional do Amapá, rio Mutum, conglomerate 02, 0°42'48"N, 51°24'48"W, 20 Feb 2009 [fl], S.V. Costa Neto et al. 2818 (HAMAB).

#### Distribution and ecology.

It is known to occur in Guyana, French Guiana, Suriname and Brazil. It is recorded for the state of Amapá, growing near river banks, in *Floresta de Terra Firme* and in periodically flooded areas called *Floresta de Várzea*.

#### Taxonomic comments.

*Passiflora amoena* belongs to P. subg. Astrophea, sect. Botryastrophea (Harms) Killip, ser. Carneae Feuillet. It is morphologically most similar to *P. fuchsiiflora*, being differentiated from the latter by its flowers with yellow to orange-yellow corona and dolabriform-triangular outer corona filaments. Both species possess pollen grains of the IV type (Mezzonato-Pires et al. 2017) due to their reticulate sexine and small lumina.

### Passiflora fuchsiiflora

Taxon classificationPlantaeMalpighialesPassifloraceae

Hemsl., Icon. Pl. 26: pl. 2553. 1898.

Figs 2C, D , 3C, D

#### Descriptions.

***Lianas***; tendrils robust, glabrous. ***Stipules*** not seen. ***Petioles*** with two glands on the terminal end of the adaxial side. ***Blades*** 13.2–15.2 × 11.2–13.5 cm, chartaceous, widely ovate, apex round to slightly emarginate, base slightly to deeply cordate, glabrous on both sides; margins non-undulate, glandular; 11–14 pairs of secondary veins, arcuate. ***Bracts*** diminute, linear-setaceous, alternate. ***Flowers*** arranged in a racemose inflorescence, hypanthium cylindrical; sepals linear-oblong, pink; petals linear-oblong, pink; corona with 3 series of filaments, dark purple, filaments of first series subdolabriform, laterally attached filaments, filaments of second series tuberculate to tuberculate-triangular, filaments of third tuberculate; operculum straight, membranous, non-tubular, included, apex fimbriate; trochlea absent on the androgynophore; ovary obovoid to oblongoid, glabrous. ***Fruits*** not seen.

#### Palynology.

Pollen grains medium-sized (ca. 38.4 µm), prolate spheroidal, 6-colporate, colpi long, narrow, three endoapertures lalongate (ca. 7.6 × 11.0 µm), unique for each pair of ectoaperture, sexine reticulate, heterobrochate; muri (ca. 1.2 µm) simple columellate, sinuous, continuous, with perforations, without high columellae, not apparent, tectum surface mostly curved, lumina slightly ornamented, small (ca. 4.0 µm diam.) (Fig. 3C, D).

#### Specimens examined.

**BRAZIL. Amazonas**: Presidente Figueiredo, Rebio Uatumã, grid of PPBio, 2°2'4"S, 60°1'30"W, 16 Apr 2007 [fl, fr], S. Sakagawa et al. 248 (INPA); Rebio Uatumã, Igarapé access to camp 2, left bank rising, 2°2'4"S, 60°1'30"W, 27 Sep 2008 [fl], S. Sakagawa and J.R.M. Ferreira 512 (INPA).

#### Distribution and ecology.

It is known to occur in Guyana, French Guiana, Suriname, Venezuela and Brazil. A single specimen collected in 1993 at the Indigenous Reserve of Yanomani, state of Amazonas, by *W. Miliken 1801*, was originally identified as *P. fuchsiiflora*. Until the present contribution, this was the only specimen of *P. fuchsiiflora* known for Brazil. Nonetheless, this specimen actually belongs to *P. balbis* Feuillet. Two specimens from the state of Amazonas, municipality of Presidente Figueiredo, at the Biological Reserve of Uatumã, are here correctly identified as *P. fuchsiiflora*. Thus, this species is recorded for the first time in Brazil based on the aforementioned specimens. In Brazil, *P. fuchsiiflora* is restricted to the state of Amazonas, growing in *Igarapé* formations.

#### Taxonomic comments.

*Passiflora fuchsiiflora* belongs to P. subg. Astrophea, sect. Botryastrophea, ser. Carneae. It possesses widely ovate and chartaceous leaf-blades, with round to slightly emarginate apex, added to the three filament series of the corona, with subdolabriform outer filaments and the two inner series with tuberculate filaments. The pollen grains possess reticulate sexine with small lumina and, for this reason, the pollen was included by Mezzonato-Pires et al. (2017) in the type IV pollen group.

### Passiflora jussieui

Taxon classificationPlantaeMalpighialesPassifloraceae

Feuillet, Journal of the Botanical Research Institute of Texas 4(2): 611, f. 1. 2010.

Figs 2E, F , 3E, F

#### Descriptions.

***Lianas***; tendrils slightly robust, glabrous to slightly puberulous. ***Stipules*** not seen. ***Petioles*** with two glands on the terminal end of the adaxial side. ***Blades*** 15–23 × 8.4–12.7 cm, coriaceous, ovate to ovate-oblong to oblong to widely oblong, apex acuminate, base truncate to round, glabrous on both sides; margins conspicuous, undulate, brown to brownish-green, glandular, with 2–3 glands; 16–23 pairs of secondary veins, arcuate. ***Bracts*** diminute, linear-setaceous, alternate. ***Flowers*** arranged in a racemose inflorescence, hypanthium cylindrical; sepals linear-oblong, greenish; petals linear-oblong, white with lilac spots; corona with 4–6 series of filaments, greenish-yellow with dark red to purplish spots, filaments of first series subdolabriform, straight, laterally attached, filaments of second, third and fourth series with linear-capitate, straight, filaments of fifth series linear-capitate to hair-like, semi-straight or reflexed, filaments of sixth series hair-like, reflexed; operculum straight, tubular, membranous, exserted, apex crenulate; trochlea absent on the androgynophore; ovary narrowly oblongoid to narrowly ovoid, densely velutine. **Fruits** not seen.

#### Palynology.

Pollen grains large-sized (ca. 67.6 µm), prolate spheroidal, 6-colporate, colpi short, narrow, three endoaperture lalongate (ca. 5.5 × 9.2 µm) unique for each pair of ectoaperture, sexine partially tectate as small pieces of remnant muri can be observed; muri (ca. 2.8 µm) duplicolumellate, columellae high, apparent, without perforations and most of the tectum surface curved or with spines (ca. 4.8 × 4.6 µm), not forming lumina. A large part of the surface is ornamented with sparsely distributed bacula and conspicuous pila (Fig. 3E, F).

#### Specimens examined.

**BRAZIL. Acre**: Cruzeiro do Sul: Reserva Extrativista do Alto Juruá, Rio Juruá, Seringal São João, placing Tapaúna, [7°37'52"S, 72°40'12"W], 14 Mar 1992 [fl], D.C. Daly et al. 7423 (INPA, UPCB). **Rondônia**: Serra do Balaterio, 7 km from the village Campo Novo, 10°35'0S, 63°39'0"W, 24 Apr 1987 [fl], C.A. Cid et al. 8915 (INPA).

#### Distribution and ecology.

It is known to occur in French Guiana, Suriname and Brazil, being also cultivated at the ORSTOM Botanical Garden in Cayenne, in the UK by R.J.R. Vanderplank (Feuillet 2010) and in the USA by L. Gilbert. It is recorded for the states of Acre (municipality of Cruzeiro do Sul) and Rondônia (municipality of Porto Velho). According to Feuillet (2010), *P. jussieui* can be found growing in lowland rainforests, whereas in Brazil, it is found in *Floresta de Terra Firme* formations, in sandy and rocky soils.

#### Taxonomic comments.

*Passiflora jussieui* belongs to P. subg. Astrophea sect. Capreolata J.M.MacDougal & Feuillet. It can be characterised by its leaf-blades with conspicuous margins, flowers with corona arranged in 4–6 filament series with dark red to purplish spots, the outer series with subdolabriform filaments, the inner series with linear-capitelate and straight filaments and the sixth series with hair-like and reflexed filaments. The pollen grains of the closely related *P. cerradensis* Sacco present semitectate exine and reticulate sexine with large and ornamented lumina; which differ greatly from the mostly non-tectate exine, not producing lumina, pollen grains of *P. jussieui*. The pollen grains of *P. jussieui* are included in the type II pollen group, while the ones of *P. cerradensis* are included in the type III pollen group (Mezzonato-Pires et al. 2017).

### Passiflora ovata

Taxon classificationPlantaeMalpighialesPassifloraceae

Jos. Martin ex DC., Prodr. 3: 322. 1828. Astrophea ovata (Jos. Martin ex DC.) M. Roem. Familiarum Naturalium Regni Vegetabilis Synopses Monographicae 2: 151. 1846.

Figs 2G, H , 3G, H

#### Descriptions.

***Lianas***; tendrils not seen. ***Stipules*** diminute, linear to linear-falcate. ***Petioles*** with two glands on the terminal end of the adaxial side. ***Blades*** 9.5–19.3 × 4.3–8.7 cm, chartaceous, oblong to obovate, apex attenuate to abruptly attenuate, base obtuse to round, glabrous on both sides; margins slightly undulate, with 4–6 glands; 22–25 pairs of secondary veins. ***Bracts*** diminute, linear to linear-falcate, alternate. ***Flowers*** solitary, hypanthium widely campanulate; sepals oblong, light green; petals oblong, white; corona with 4–6 series of filaments, filaments of first series dolabriform, with apex narrowly linear, margins slightly undulate, yellow below the inflated portion of the filaments, orange-yellow in the inflated portion, filaments of second series linear, filaments of third and fourth series with hair-like, filaments of fifth and sixth series with hair-like, reflexed; operculum straight, tubular, exserted, filamentous with a fimbriate apex, papillose; trochlea absent on the androgynophore; ovary obovoid to oblong-ovoid, densely tomentose. ***Fruits*** 5.5 × 3.5 cm, ellipsoid, glabrous.

#### Palynology.

Pollen grains large-sized (ca. 56.6 µm), prolate spheroidal, 6-colporate, colpi short, narrow, three endoaperture lalongate (ca. 10.0 × 15.5 µm) unique for each pair of ectoaperture, sexine reticulate, heterobrachate; muri (ca. 1.9 µm) duplicolumellate, sinuous, continuous, without perforations, without high columellae, not apparent, tectum surface mostly slightly curved, lumina slightly ornamented with pila, large (ca. 13.4 µm diam.) (Fig. 3G and H).

#### Specimens examined.

**BRAZIL. Acre**: Acrelândia, PAE Porto Dias (placing Bibi), 9°49'40"S, 66°53'0"W, 09-15 Nov 2006 [fl, fr], F. Obermuller et al. 102 (RB). **Amazonas**: São Gabriel da Cachoeira: Highway BR-307, SGC-Cucuí km 50, [0°16'25"N, 66°39'35"W], 27 Oct 2008 [fl], S. Sakagawa et al. 668 (INPA); Highway BR-307, SGC-Cucuí km 50, [0°15'49"N, 66°40'56"W], 22 Apr 2008, R.L. Assis et al. 93 (INPA).

#### Distribution and ecology.

It is known to occur in French Guiana, Venezuela and Brazil. It is recorded here for the states of Acre (municipality of Acrelândia) and Amazonas (municipality of São Gabriel da Cachoeira), growing in *Floresta Ombrófila Densa* formations, along roadsides, reaching up to 10 m high.

#### Taxonomic comments.

*Passiflora ovata* belongs to P. subg. Astrophea sect. Pseudoastrophea (Harms) Killip. It can be easily differentiated due to its oblong to obovate leaf-blades, glands restricted to the abaxial side of the petiole near the blade, flowers with widely campanulate hypanthium, and dolabriform filaments on the first corona series. According to Escobar (1990), the straight and tubular operculum of *P. ovata*, probably favours hummingbird pollination. The most morphologically similar species is *P. costata*, which can be easily distinguished from *P. ovata* by the presence of a trochlea in the androgynophore, operculum declinate at base with a straight upper part and corona with non-reflexed filaments in the inner series. According to Mezzonato-Pires et al. (2017), *P. ovata* is included in the type III pollen group, due to its reticulate sexine and large lumina.

### Passiflora plumosa

Taxon classificationPlantaeMalpighialesPassifloraceae

Feuillet & Cremers, Proceedings of the Koninklijke Nederlandse Akademie van Wetenschappen, Series C: Biological and Medical Sciences 87(3): 381, f. 2. 1984.

Fig. 2I, J

#### Descriptions.

***Lianas***; tendrils thin, glabrous. ***Stipules*** diminute, linear. ***Petioles*** with two glands on the terminal end of the adaxial side. ***Blades*** 14.2–22 × 6.3–8.5 cm, membranous, oblong, apex attenuate, base obtuse, glabrous on both sides; margins inconspicuous, with two glands at base; 13–19 pairs of secondary veins, arcuate. ***Bracts*** diminute, deltoid, alternate. ***Flowers*** solitary or arranged in racemose inflorescences, hypanthium cylindrical to slightly funnelform; sepals linear-oblong; petals linear-oblong; corona with 4 series of filaments, white, filaments of first series liguliform, filaments of second series linear or liguliform, filaments of third and fourth series liguliform, plumose, reflexed or not; operculum straight, tubular exserted, apex crenulate; trochlea absent in the androgynophore; ovary oblongoid, densely velutine. ***Fruits*** not seen.

#### Palynology.

Pollen grains not seen.

#### Specimens examined.

**BRAZIL. Amazonas**: Itapiranga: rio Uatumã, the left bank, in front of the Rio Pitinga, [2°44'56"S, 58°1'19"W], 27 Aug 1979 [fl], C.A.Cid et al. 561 (MG); Presidente Figueiredo, Rebio Uatumã, 1°00’S, 59°00"W, grid of PPBio s.n., portion L3/500, [2°2'4"S, 60°1'30"W], 11 Jul 2008 [fl], F.A. Carvalho 31UAT (INPA).

#### Distribution and ecology.

The species was hitherto known to occur exclusively in its type locality, in the central-southern forests of French Guiana. Two specimens were identified by the authors, occurring in the state of Amazonas, municipalities of Itapiranga and Presidente Figueiredo. The new-found specimens were collected growing in *Floresta Ombrófila Densa* and *Floresta de Terra Firme* formations, on river edges with clay soil, at 600 m elevation.

#### Taxonomic comments.

*Passiflora plumosa* belongs to P. subg. Astrophea sect. Leptopoda Killip *ex* Feuillet & Cremers. It can be differentiated by its glabrous and membranous leaf-blades, corona with four series of filaments, all of them being liguliform in shape and the second and fourth or the third and fourth series with reflexed filaments. The apex of the operculum of *P. plumosa* can easily distinguish this species from *P. cauliflora* Harms and *P. cerradensis*, due to its crenulate apex.

### Passiflora quelchii

Taxon classificationPlantaeMalpighialesPassifloraceae

N.E. Br., Transactions of the Linnean Society of London, Botany 6: 31, pl. 3. 1901.

Figs 2K, L , 3H, I

#### Descriptions.

***Shrubs***; tendrils absent. ***Stipules*** diminute, narrowly triangular, glabrous. ***Petioles*** with two glands on the terminal end of the adaxial side. ***Blades*** 11.3–18.8 × 2.6–4.8 cm, chartaceous, narrowly oblong to oblong-lanceolate, apex acute, base cuneate, glabrous on both sides; margins strongly undulate, with ca. three glands; 29–33 pairs of secondary veins, arcuate, conspicuous. ***Bracts*** diminute, triangular, verticillate, with marginal glands. ***Flowers*** arranged in racemose inflorescences, hypanthium cylindrical; sepals oblong-lanceolate; petals oblong-lanceolate; corona with 2 series of filaments, filaments of first series dolabriform, filaments of second series hair-like, apex entire or bifid, straight; operculum straight, non-tubular, included, filamentous with a fimbriate apex, 3.4–3.5 cm long; trochlea absent on the androgynophore; ovary oblongoid, glabrous. ***Fruits*** ca. 3.11 × 2.93 cm, orbicular, glabrous, green.

#### Palynology.

Pollen grains medium-sized (ca. 47.8 µm), prolate spheroidal, 6-colporate, colpi short, narrow, three endoaperture lalongate (ca. 8.7 × 17.0 µm) unique for each pair of ectoaperture, sexine reticulate, heterobrochate; muri (ca. 2.0 µm) duplicolumellate, sinuous, continuous, without perforations, columellae high, apparent, tectum surface mostly slightly curved, lumina ornamented with pila, large (ca. 15.2 µm diam.) (Fig. 3H, I).

#### Specimens examined.

**BRAZIL. Roraima**: Cantá, Serra Grande, 2°32'53"S, 60°47'10"W, 554 m, 12 Nov 2014 [fl], R.C. Forzza et al. 8321 (RB).

#### Distribution and ecology.

Found in savannahs from southwestern Guyana, being here recorded in the state of Roraima, municipality of Cantá, in the Serra Grande inselberg, around 554 m elevation. Beside the type specimens and the one recorded for Brazil, *P. quelchii* is only known by another specimen (i.e., *Graham 342*) from the Ireng District in Guyana (Escobar 1990).

#### Taxonomic comments.

*Passiflora quelchii* belongs to P. subg. Astrophea sect. Capreolata. It can be distinguished by its shrubby habit and leaf-blades with undulate margins and inconspicuous *in sicco*. The included, non-tubular, filamentous operculum with fimbriate apex is characteristic to *P. quelchii* and easily differentiates it from closely related species. *Passiflora quelchii* possess type III pollen grains, with reticulate sexine forming large lumina (Mezzonato-Pires et al. 2017).

### Passiflora tessmannii

Taxon classificationPlantaeMalpighialesPassifloraceae

Harms, Notizbl. Bot. Gart. Berlin-Dahlem 9: 978. 1926.

Figs 2M, N , 3K, L

#### Descriptions.

***Lianas***; tendrils absent. Stipules not seen. ***Petioles*** with two glands on the terminal end of the adaxial side. ***Blades*** 6.2–11 × 4–8 cm, chartaceous, elliptic to ovate to oblong-elliptic, apex emarginate, mucronate, base cuneate to round, abaxially puberulous, adaxially glabrous, except for the midvein; margins revolute, non-undulate, glands not seen; 10–14 pairs of secondary veins, arcuate. ***Bracts*** diminute, triangular, alternate. ***Flowers*** arranged in pairs, hypanthium campanulate-cylindric; sepals linear-lanceolate, green; petals linear-lanceolate, white; corona with 2 series of filaments, white at base, yellowing at mid length, with vinaceous spots at apex, filaments of first series linear, apex widely falcate, attenuate, filaments of second series subulate, apex acute; operculum straight, with irregular slits, non-tubular, included, filaments with fimbriate apex, papillose; androgynophore with trochlea; ovary oblongoid to obovoid, velutine to pilose, with hairs restricted to the veins. ***Fruits*** 6.3–6.4 × 2.3–2.4 cm, ellipsoid, puberulous.

#### Palynology.

Pollen grains medium-sized (ca. 46.0 µm), oblate spheroidal, 6-colporate, colpi long, narrow, three endoaperture lalongate (ca. 4.3 × 20.4 µm) unique for each pair of ectoaperture, sexine reticulate, heterobrochate; muri (ca. 1.0 µm) simple columellate, sinuous, continuous, with perforations, without high columellae, not apparent, tectum surface slightly curved, lumina not ornamented, small (ca. 2.4 µm diam.) (Fig. 3K, L).

#### Specimens examined.

**BRAZIL. Amazonas**: Manaus, Igarapé do Crespo, [3°6'7"S, 60°1'30"W], 04 Sep 1945 [fl], A. Ducke 1749 (IAN, NYBG, R); Itacoatiara-Manaus, Reserva Florestal Ducke, km 26, 2°53'0"S, 59°58'0"W, 10 Oct 1995 [bt, fl], C.A. Sothers and E.da C. Pereira 612 (INPA, MBM, MG, UEC); km 26, 2°53'0"S, 59°58'0"W, 27 Nov 1996, [bt, fl], M.J.G. Hopkins et al. 1609 (IAN, INPA, NYBG, SP, UB); next to road the Acará, [3°8'35"S, 58°26'39"W], 19 Dec 1997 [bt, fl], M.A.D. Souza and M.J.G. Hopkins 514 (INPA, SPF).

#### Distribution and ecology.

It is known for Ecuador and Peru, being herein recorded for the state of Amazonas, municipality of Manaus, localities of Igarapé do Crespo and Adolpho Ducke Forest Reserve. It is found growing in lowland forests and *Terra Firme* and *Campinarana* formations, in sandy soils, reaching 18 m.

#### Taxonomic comments.

*Passiflora tessmannii* belongs to P. subg. Astrophea sect. Pseudoastrophea. Until the present study, the Brazilian specimens of *P. tessmannii* were erroneously identified as *P. hexagonocarpa*, due to conflicting taxonomic characters. This confusion caused this species to be treated as *P. hexagonocarpa* in the Flora da Reserva Ducke (Hopkins and Sousa 1999). *Passiflora tessmannii* possesses conical trochlea with undulated margins, corona clearly composed of two series, outer filaments linear in shape, with apex widely falcate and attenuate and inner filaments subulate. The pollen grains possess reticulate sexine with small lumina, similarly to most species of P. subg. Astrophea sect. Pseudoastrophea and, according to Mezzonato-Pires et al. (2017), the pollen is included in the type IV pollen group.

## Supplementary Material

XML Treatment for Passiflora amoena

XML Treatment for Passiflora fuchsiiflora

XML Treatment for Passiflora jussieui

XML Treatment for Passiflora ovata

XML Treatment for Passiflora plumosa

XML Treatment for Passiflora quelchii

XML Treatment for Passiflora tessmannii

## References

[B1] Aguirre-Morales AC Bonilla-Morales MM Rojas A (2016) *Passiflora gironensis* a new species of Passiflora subgenus Astrophea (Passifloraceae) from Santander, Colombia. Phytotaxa 280(3): 278–284. http://dx.doi.org/10.11646/phytotaxa.280.3.6

[B2] Erdtman G (1952) Pollen morphology and plant taxonomy angiosperms. Almqvist & Wiksells Press, Stockholm, 539 pp.

[B3] Escobar LA (1990) Una Revision Taxonomica de *Passiflora* subgenero *Astrophea*, Passifloraceae. PhD Thesis, Universidad de Antioquia.

[B4] Feuillet C (2010) Folia taxonomica 18. The status of *Passiflora citrifolia* and a new species in subgenus Astrophea (Passifloraceae), *Passiflora jussieu*. Journal of the Botanical Research Institute of Texas 4: 609–614.

[B5] Feuillet C MacDougal J (2003) A new infrageneric classification of *Passiflora* L. (Passifloraceae). *Passiflora*: The Journal & Newsletter of Passiflora Society International 13: 34–38.

[B6] Feuillet C MacDougal J (2007) Passifloraceae. In: Kubitzki K (Ed.) The Families and Genera of Vascular Plants. Springer-Verlag, 270–280. https://doi.org/10.1007/978-3-540-32219-1_35

[B7] Flora do Brasil 2020 (2017) *Passiflora* Jardim Botânico do Rio de Janeiro. http://floradobrasil.jbrj.gov.br/reflora/floradobrasil/FB86373 [accessed 14.07.2017]

[B8] Hopkins MJG Souza MAD (1999) Passifloraceae. In: Ribeiro JES Hopkins MJG Vicentini A Sothers CA Costa MAS Brito JM Souza MAD Martins LHP Lohmann LG Assunção PACL Pereira EC Silva CF Mesquita MR Procópio LC (Eds) Flora da Reserva Ducke: Guia de Identificação das Plantas Vasculares de uma Floresta de Terra-Firme na Amazônia Central. INPA, Manaus, 299–306.

[B9] Killip EP (1938) The American species of Passifloraceae. Field Museum of Natural History, Botanical Series 19: 1–613. https://doi.org/10.5962/bhl.title.2269

[B10] Krosnick SE Ford AJ Freudenstein JV (2009) Taxonomic revision of Passiflora subgenus Tetrapathea including the monotypic genera *Hollrungia* and *Tetrapathea* (Passifloraceae), and a new species of *Passiflora*. Systematic Botany 34: 375–385. https://doi.org/10.1600/036364409788606343

[B11] Melhem TS Cruz-Barros MAV Corrêa AMS Makino-Watanabe H Silvestre-Capelato MSF Gonçalves-Esteves VL (2003) Variabilidade polínica em plantas de Campos de Jordão. Boletim do Instituto de Botânica (São Paulo) 16: 9–104.

[B12] Mezzonato-Pires AC Gonçalves-Esteves V Bernacci LC (2016) A new species of Passiflora subgenus Astrophea (Passifloraceae) from the Brazilian Amazon. Phytotaxa 288: 077–084. https://doi.org/10.11646/phytotaxa.288.1.8

[B13] Mezzonato-Pires AC Mendonça CBF Milward-de-Azevedo MA Gonçalves-Esteves V (2017) The systematic value of pollen morphology of Passiflora subgenus Astrophea (Passifloraceae). Phytotaxa 298(1): 1–19. https://doi.org/10.11646/phytotaxa.298.1.1

[B14] Punt W Blackmore S Nilsson S Le Thomas A (2007) Glossary of pollen and spore terminology. Review of Paleobotany and Palynology 143: 1–81. https://doi.org/10.1016/j.revpalbo.2006.06.008

[B15] Radford AE Dickison WC Massey JR Bell CR (1974) Vascular Plant Systematics. Harper & Row Publishers, New York, 891 pp.

[B16] Ulmer T MacDougal JM (2004) Passiflora: Passionflowers of the World. Timber Press, Cambridge, 430 pp.

